# (*S*)-6-Methyl-∊-caprolactone

**DOI:** 10.1107/S1600536808004583

**Published:** 2008-02-20

**Authors:** Maxime A. Siegler, Huub Kooijman, Anthony L. Spek

**Affiliations:** a Bijvoet Center for Biomolecular Research, Crystal and Structural Chemistry, Utrecht University, Padualaan 8, 3584 CH Utrecht, The Netherlands

## Abstract

The chiral title compound, C_7_H_12_O_2_, a lactone derivative, features a seven-membered ring that adopts a chair conformation. The crystal structure is stabilized by weak C—H⋯O inter­actions occurring in the (100) plane. The absolute configuration was assigned on the basis of the enantioselective synthesis.

## Related literature

For related literature, see: van As *et al.* (2005 ▶); van Buijtenen *et al.* (2006 ▶). For details of the synthesis, see: van As *et al.* (2007 ▶). For geometry, see: Cremer & Pople (1975 ▶).
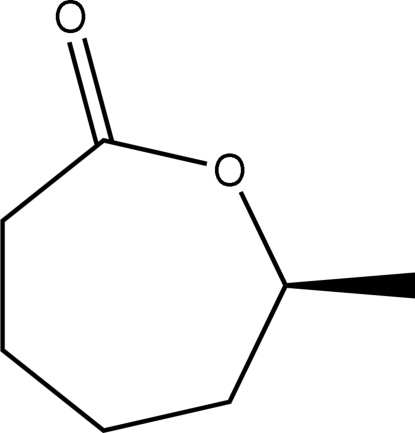


## Experimental

### 

#### Crystal data

C_7_H_12_O_2_
*M* *_r_* = 128.17
Monoclinic, 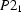

*a* = 6.757 (2) Å
*b* = 7.577 (2) Å
*c* = 7.586 (2) Å
β = 110.949 (13)°
*V* = 362.71 (17) Å^3^
*Z* = 2
Mo *K*α radiation
μ = 0.08 mm^−1^
*T* = 150 (2) K
0.35 × 0.15 × 0.10 mm

#### Data collection

Nonius KappaCCD diffractometer
Absorption correction: none
10010 measured reflections
889 independent reflections
862 reflections with *I* > 2σ(*I*)
*R*_int_ = 0.041

#### Refinement

*R*[*F* ^2^ > 2σ(*F* ^2^)] = 0.027
*wR*(*F* ^2^) = 0.072
*S* = 1.10
889 reflections
83 parameters
1 restraint
H-atom parameters constrained
Δρ_max_ = 0.12 e Å^−3^
Δρ_min_ = −0.16 e Å^−3^

### 

Data collection: *COLLECT* (Nonius, 1998 ▶); cell refinement: *DENZO* (Otwinowski & Minor, 1997 ▶); data reduction: *DENZO*; program(s) used to solve structure: *SHELXS86* (Sheldrick, 2008 ▶); program(s) used to refine structure: *SHELXL97* (Sheldrick, 2008 ▶); molecular graphics: *PLATON* (Spek, 2003 ▶); software used to prepare material for publication: *PLATON* and *Mercury* (Macrae *et al.*, 2006 ▶).

## Supplementary Material

Crystal structure: contains datablocks I, global. DOI: 10.1107/S1600536808004583/tk2248sup1.cif

Structure factors: contains datablocks I. DOI: 10.1107/S1600536808004583/tk2248Isup2.hkl

Additional supplementary materials:  crystallographic information; 3D view; checkCIF report

## Figures and Tables

**Table 1:** Short-contact C—H⋯O interactions (Å, °) found in the (100) plane

C—H⋯O	C—H	H⋯O	C⋯O	C—H⋯A
C2—H2*A*⋯O1^i^	0.99	2.67	3.573 (2)	152
C5—H5*A*⋯O1^ii^	0.99	2.64	3.616 (2)	166
C5—H5*B*⋯O1^ii^	0.99	2.63	3.601 (2)	168
C6—H6⋯O1^i^	1.00	2.54	3.466 (2)	154

Symmetry codes: (i) 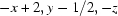; (ii) 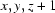.

**Fig. 1.**
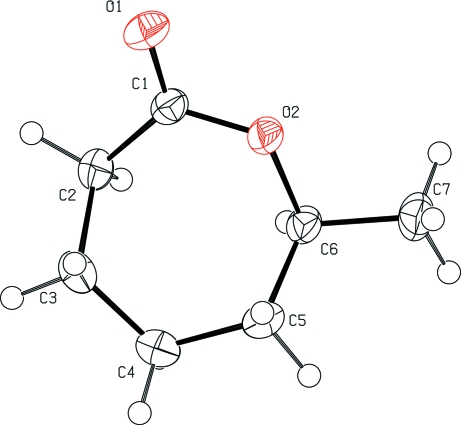
Molecular structure of (I) showing atom labelling and displacement ellipsoids at the 50% probability level.

**Fig. 2.**
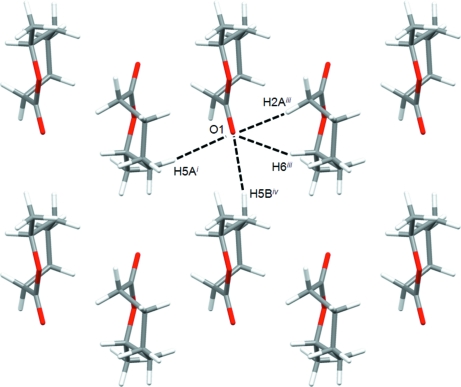
The packing of one layer found in the crystal structure of (I) viewed down the a* direction. Molecules are connected by short C–H···O contacts in the (1 0 0) plane. Only one set of short contacts (dashed lines) is shown for clarity. The symmetry codes are: (i) 2 - x, -1/2 + y, -z; (iii) 2 - x, 1/2 + y, -z; (iv) x, y, z - 1.

## supplementary crystallographic information

### Comment

The enantiomers (*R*)- and (*S*)-6-methyl-ε-caprolactone (6-MeCL) have been recently used as monomer units for chiral oligomerization and chiral polymerization reactions (van As *et al.*, 2005; van Buijtenen *et al.*, 2006). A new two-step enantioselective synthesis of (*R*)- and (S)-6-MeCL from a racemic mixture of 6-MeCL has been described recently (van As *et al.*, 2007). The present paper describes the crystal structure of (S)-6-MeCL, (I).

The structure of (I) (Fig. 1) was solved in the non-centrosymmetric space group *P*2_1_ with *Z'* = 1. The stereochemistry at the chiral center, C6, was assigned S based on the enantioselective synthesis of (S)-6-MeCL, which was reported to yield an enantiomeric excess greater than 99% (van As *et al.*, 2007).

The seven-membered ring (O2/C1—C6) adopts a chair conformation with puckering parameters: *Q_2_* = 0.453 (2) Å, *φ_2_* = 130.6 (2)°, *Q_3_* = 0.653 (2) Å, *φ_3_* = 102.6 (2)° and with a total puckering amplitude *Q* = 0.795 (2)Å (Cremer & Pople, 1975).

The crystal structure (I) is stabilized by weak C–H···O interactions (Table 1) with the four shortest contacts involving the O1 atom. These short contacts occur between molecules in the (1 0 0) plane, Fig. 2.

### Experimental

Details about the synthesis of (*S*)-6-methyl-ε-caprolactone have been given in a previous paper (van As *et al.*, 2007).

### Refinement

In the absence of significant anomalous scattering effects, XXX Friedel pairs were merged prior to the refinement. The H atoms were found in difference Fourier maps and subsequently placed at calculated positions with C–H = 0.99–1.00 Å, and with *U*_iso_(H) = 1.2 or 1.5 times *U*_eq_(carrier C).

### Crystal data

**Table:**

C_7_H_12_O_2_	*F*_000_ = 140
*M**_r_* = 128.17	*D*_x_ = 1.174 Mg m^−^^3^
Monoclinic, *P*2_1_	Mo *K*α radiation λ = 0.71073 Å
Hall symbol: P 2yb	Cell parameters from 7835 reflections
*a* = 6.757 (2) Å	θ = 1.0–27.5º
*b* = 7.577 (2) Å	µ = 0.08 mm^−^^1^
*c* = 7.586 (2) Å	*T* = 150 (2) K
β = 110.949 (13)º	Prism, colourless
*V* = 362.71 (17) Å^3^	0.35 × 0.15 × 0.10 mm
*Z* = 2	

### Data collection

**Table:**

Nonius KappaCCD diffractometer	862 reflections with *I* > 2σ(*I*)
Radiation source: rotating anode	*R*_int_ = 0.041
Monochromator: graphite	θ_max_ = 27.5º
*T* = 150(2) K	θ_min_ = 3.5º
φ and ω scans	*h* = −8→8
Absorption correction: none	*k* = −9→9
10010 measured reflections	*l* = −9→9
889 independent reflections	

### Refinement

**Table:**

Refinement on *F*^2^	Secondary atom site location: difference Fourier map
Least-squares matrix: full	Hydrogen site location: difference Fourier map
*R*[*F*^2^ > 2σ(*F*^2^)] = 0.027	H-atom parameters constrained
*wR*(*F*^2^) = 0.072	*w* = 1/[σ^2^(*F*_o_^2^) + (0.0431*P*)^2^ + 0.0282*P*] where *P* = (*F*_o_^2^ + 2*F*_c_^2^)/3
*S* = 1.10	(Δ/σ)_max_ < 0.001
889 reflections	Δρ_max_ = 0.12 e Å^−^^3^
83 parameters	Δρ_min_ = −0.16 e Å^−^^3^
1 restraint	Extinction correction: none
Primary atom site location: structure-invariant direct methods	Absolute structure: known chirality of atom C6(S)

### Special details

**Table:**

Geometry. All e.s.d.'s (except the e.s.d. in the dihedral angle between two l.s. planes) are estimated using the full covariance matrix. The cell e.s.d.'s are taken into account individually in the estimation of e.s.d.'s in distances, angles and torsion angles; correlations between e.s.d.'s in cell parameters are only used when they are defined by crystal symmetry. An approximate (isotropic) treatment of cell e.s.d.'s is used for estimating e.s.d.'s involving l.s. planes.
Refinement. Refinement of *F*^2^ against ALL reflections. The weighted *R*-factor *wR* and goodness of fit *S* are based on *F*^2^, conventional *R*-factors *R* are based on *F*, with *F* set to zero for negative *F*^2^. The threshold expression of *F*^2^ > 2σ(*F*^2^) is used only for calculating *R*-factors(gt) *etc*. and is not relevant to the choice of reflections for refinement. *R*-factors based on *F*^2^ are statistically about twice as large as those based on *F*, and *R*- factors based on ALL data will be even larger.

### Fractional atomic coordinates and isotropic or equivalent isotropic displacement parameters (Å^2^)

**Table:**

	*x*	*y*	*z*	*U*_iso_*/*U*_eq_	
O1	1.03545 (16)	0.06828 (15)	−0.16671 (13)	0.0355 (3)	
O2	1.11573 (13)	0.04935 (14)	0.13758 (12)	0.0288 (2)	
C1	0.9754 (2)	0.03155 (18)	−0.03896 (16)	0.0258 (3)	
C2	0.7534 (2)	−0.0285 (2)	−0.07029 (18)	0.0301 (3)	
H2A	0.7594	−0.1422	−0.0042	0.036*	
H2B	0.6778	−0.0491	−0.2069	0.036*	
C3	0.6274 (2)	0.1054 (2)	0.0003 (2)	0.0356 (3)	
H3A	0.6603	0.2261	−0.0311	0.043*	
H3B	0.4740	0.0849	−0.0669	0.043*	
C4	0.6759 (2)	0.0938 (2)	0.2120 (2)	0.0365 (3)	
H4A	0.6389	−0.0260	0.2421	0.044*	
H4B	0.5841	0.1787	0.2461	0.044*	
C5	0.9066 (2)	0.1315 (2)	0.33415 (18)	0.0303 (3)	
H5A	0.9423	0.2528	0.3071	0.036*	
H5B	0.9205	0.1274	0.4685	0.036*	
C6	1.06592 (19)	0.00512 (19)	0.30521 (16)	0.0267 (3)	
H6	1.0098	−0.1181	0.2938	0.032*	
C7	1.2785 (2)	0.0148 (3)	0.46522 (19)	0.0403 (4)	
H7A	1.3333	0.1356	0.4759	0.060*	
H7B	1.3783	−0.0660	0.4396	0.060*	
H7C	1.2615	−0.0190	0.5837	0.060*	

### Atomic displacement parameters (Å^2^)

**Table:**

	*U*^11^	*U*^22^	*U*^33^	*U*^12^	*U*^13^	*U*^23^
O1	0.0442 (5)	0.0406 (6)	0.0260 (5)	0.0001 (5)	0.0177 (4)	0.0018 (4)
O2	0.0248 (4)	0.0400 (6)	0.0219 (4)	−0.0028 (4)	0.0087 (3)	0.0022 (4)
C1	0.0305 (6)	0.0245 (6)	0.0221 (6)	0.0029 (5)	0.0093 (5)	−0.0007 (5)
C2	0.0272 (6)	0.0341 (7)	0.0255 (6)	−0.0021 (5)	0.0050 (5)	−0.0041 (6)
C3	0.0242 (6)	0.0431 (9)	0.0365 (7)	0.0058 (6)	0.0074 (5)	0.0002 (7)
C4	0.0298 (6)	0.0451 (9)	0.0382 (7)	0.0031 (6)	0.0166 (5)	−0.0030 (7)
C5	0.0360 (7)	0.0322 (7)	0.0254 (6)	−0.0015 (6)	0.0143 (5)	−0.0026 (5)
C6	0.0265 (6)	0.0330 (7)	0.0202 (6)	−0.0027 (5)	0.0079 (5)	0.0028 (5)
C7	0.0311 (6)	0.0598 (11)	0.0252 (6)	−0.0040 (7)	0.0043 (5)	0.0070 (7)

### Geometric parameters (Å, °)

**Table:**

O1—C1	1.2095 (16)	C4—H4A	0.9900
O2—C1	1.3428 (15)	C4—H4B	0.9900
O2—C6	1.4659 (14)	C5—C6	1.5140 (19)
C1—C2	1.5028 (18)	C5—H5A	0.9900
C2—C3	1.539 (2)	C5—H5B	0.9900
C2—H2A	0.9900	C6—C7	1.5152 (18)
C2—H2B	0.9900	C6—H6	1.0000
C3—C4	1.523 (2)	C7—H7A	0.9800
C3—H3A	0.9900	C7—H7B	0.9800
C3—H3B	0.9900	C7—H7C	0.9800
C4—C5	1.5288 (19)		
			
C1—O2—C6	122.88 (10)	C5—C4—H4B	108.6
O1—C1—O2	117.15 (12)	H4A—C4—H4B	107.6
O1—C1—C2	123.02 (11)	C6—C5—C4	114.69 (12)
O2—C1—C2	119.82 (11)	C6—C5—H5A	108.6
C1—C2—C3	112.99 (12)	C4—C5—H5A	108.6
C1—C2—H2A	109.0	C6—C5—H5B	108.6
C3—C2—H2A	109.0	C4—C5—H5B	108.6
C1—C2—H2B	109.0	H5A—C5—H5B	107.6
C3—C2—H2B	109.0	O2—C6—C5	112.13 (11)
H2A—C2—H2B	107.8	O2—C6—C7	103.76 (10)
C4—C3—C2	113.05 (12)	C5—C6—C7	111.89 (12)
C4—C3—H3A	109.0	O2—C6—H6	109.6
C2—C3—H3A	109.0	C5—C6—H6	109.6
C4—C3—H3B	109.0	C7—C6—H6	109.6
C2—C3—H3B	109.0	C6—C7—H7A	109.5
H3A—C3—H3B	107.8	C6—C7—H7B	109.5
C3—C4—C5	114.64 (11)	H7A—C7—H7B	109.5
C3—C4—H4A	108.6	C6—C7—H7C	109.5
C5—C4—H4A	108.6	H7A—C7—H7C	109.5
C3—C4—H4B	108.6	H7B—C7—H7C	109.5
			
C6—O2—C1—O1	−178.35 (12)	C3—C4—C5—C6	−61.49 (19)
C6—O2—C1—C2	2.78 (18)	C1—O2—C6—C5	−68.78 (16)
O1—C1—C2—C3	−112.90 (16)	C1—O2—C6—C7	170.29 (13)
O2—C1—C2—C3	65.90 (16)	C4—C5—C6—O2	80.45 (14)
C1—C2—C3—C4	−81.25 (16)	C4—C5—C6—C7	−163.43 (12)
C2—C3—C4—C5	61.39 (19)		

### Table 1 Short-contact C—H···O interactions (Å, °) found in the (100) plane

**Table:**

C—H···O	C—H	H···O	C···O	C—H···A
C2—H2A···O1^i^	0.99	2.67	3.573 (2)	152
C5—H5A···O1^ii^	0.99	2.64	3.616 (2)	166
C5—H5B···O1^ii^	0.99	2.63	3.601 (2)	168
C6—H6···O1^i^	1.00	2.54	3.466 (2)	154

Symmetry codes: (i) -x+2, y-1/2, -z; (ii) x, y, z+1.
